# HOX gene dysregulation in head and neck squamous cell carcinoma: mechanisms, clinical relevance, and future perspectives

**DOI:** 10.3389/fonc.2026.1807003

**Published:** 2026-06-15

**Authors:** Norma Carolina Hernandez-Bautista, Claudia Altamirano-Torres, Jose Manuel Vazquez-Guillen, Reyes S. Tamez-Guerra, Cristina Rodríguez-Padilla, Diana Resendez-Perez

**Affiliations:** 1 Universidad Autónoma de Nuevo León, Facultad de Ciencias Biológicas, Departamento de Biología Celular y Genética, San Nicolás de los Garza, Nuevo León, Mexico; 2 Universidad Autónoma de Nuevo León, Facultad de Ciencias Biológicas, Laboratorio de Inmunología y Virología, San Nicolás de los Garza, Nuevo León, Mexico

**Keywords:** head and neck cancer, homeobox, homeotic genes, HOX genes, squamous cell carcinoma

## Abstract

Accumulated genomic and transcriptomic evidence in head and neck squamous cell carcinoma (HNSCC) has revealed widespread molecular alterations associated with tumor initiation, progression, and therapeutic resistance. Among these, deregulation of *HOX* genes has emerged as a prominent feature of cancer biology. *HOX* genes encode a highly-conserved family of transcription factors that regulate essential cellular processes, including proliferation, differentiation, migration, and survival, all of which are directly relevant to tumor development. However, their clinical exploitation as reliable biomarkers or therapeutic targets remains limited, underlining the need to identify functionally relevant molecular drivers. This mini-review provides an updated overview of *HOX* gene dysregulation in HNSCC, highlighting their context-dependent roles as oncogenes or tumor suppressors. We synthesize current evidence on the molecular mechanisms and regulatory networks governing *HOX* activity and evaluate their emerging clinical relevance as biomarkers and therapeutic targets. Finally, we identify critical knowledge gaps and propose future directions to advance *HOX*-focused translational research in HNSCC.

## 1 Introduction

**Figure 1:**
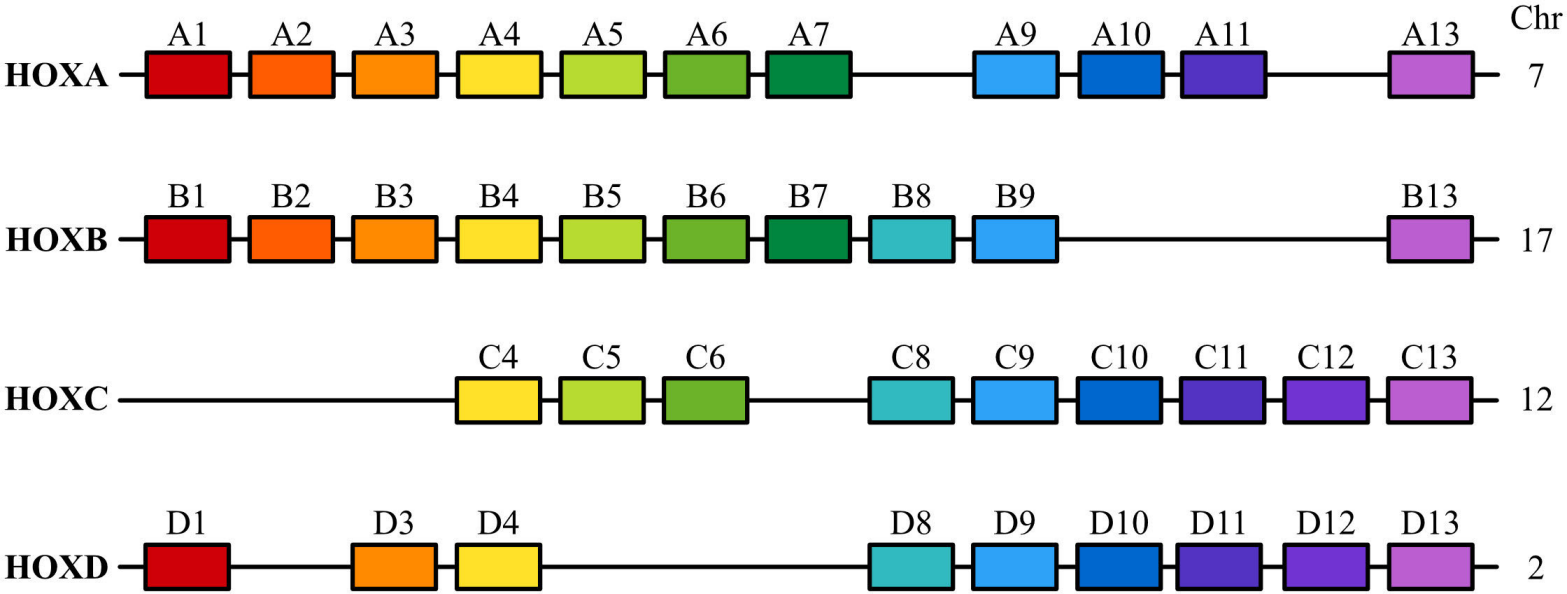
Schematic representation of the genomic organization of the 39 human *HOX* genes into four clusters (*HOXA*-*D*) and their respective chromosomal locations.

*HOX* genes encode a highly-conserved family of transcription factors that function as master regulators of embryonic development by establishing positional identity along the anteroposterior body axis (1, 2). The proteins encoded by *HOX* genes contain a conserved homeodomain that mediates DNA binding and enables tissue-specific transcriptional regulation (3). In humans, 39 *HOX* genes are organized into four clusters (*HOXA*, *HOXB*, *HOXC*, and *HOXD*), located on chromosomes 7, 17, 12, and 2, respectively (**Figure 1**). Each cluster comprises between 9 and 11 genes, which are classified based on sequence similarity and their relative position within the cluster. These genes exhibiting spatial and temporal colinear expression patterns that collectively establish a molecular code governing tissue patterning and organogenesis (4, 5). Through this activity, *HOX* genes control essential cellular processes, including migration, differentiation, proliferation, and apoptosis during development. In adult tissues, their expression is largely silenced or restricted to specific cell populations, particularly stem and progenitor cells, where they contribute to tissue homeostasis (6, 7). However, aberrant reactivation or dysregulation of *HOX* gene expression has been associated with a wide range of malignancies. Depending on the cellular context, *HOX* genes may function as oncogenes or tumor suppressors, contributing to diverse biological and clinical outcomes (8).

Head and neck squamous cell carcinoma (HNSCC) arises from the mucosal epithelia of the upper aerodigestive tract, including the oral cavity, pharynx, and larynx, and accounts for approximately 90% of all head and neck cancers (HNC) (9–12). According to GLOBOCAN 2022, HNSCC is the seventh most common cancer worldwide, with approximately 940,000 new cases and 480,000 deaths annually. Major etiological risk factors include tobacco use, alcohol consumption, and human papillomavirus (HPV) infection (13–15). Despite advances in treatment, prognosis remains poor, largely due to late-stage diagnosis and marked molecular heterogeneity. These challenges underline the need for improved molecular characterization to support early detection and development of targeted therapeutic strategies.

From a developmental perspective, head and neck epithelia arise from highly specialized embryonic structures requiring precise spatial patterning and positional identity (16). The maintenance of this identity in adult tissues is essential for homeostasis, and its disruption may contribute to malignant transformation (17). In this context, HNSCC provides a relevant model to investigate how alterations in *HOX* gene regulation may influence tumor initiation, progression, and phenotypic plasticity.

Despite increasing evidence supporting the involvement of *HOX* gene networks in HNSCC biology, their mechanisms of deregulation, context-dependent roles, and clinical relevance remain incompletely understood (18). The differential regulation of *HOX* clusters across anatomical subsites and molecular subtypes of HNSCC, as well as their contribution to tumor progression, require further investigation. This mini-review summarizes current knowledge on *HOX* gene dysregulation in HNSCC, highlighting their context-dependent roles and clinical relevance. We discuss key regulatory mechanisms, identify critical knowledge gaps, and outline future research directions.

## 2 Dysregulation of *HOX* genes in HNSCC

Although *HOX* gene dysregulation is widely reported across multiple cancer types, their precise functional contribution to tumor pathogenesis remains incompletely defined. In adult tissues, aberrant *HOX* gene expression can reactivate embryonic developmental programs, thereby disrupting normal cellular growth and differentiation, and contributing to tumorigenesis (19, 20). However, the effects of *HOX* genes deregulation are highly context-dependent, varying according to cell type, anatomical site, and tumor origin. In some contexts, *HOX* genes promote tumor progression, whereas in others specific *HOX* clusters exhibit tumor-suppressive functions (21).

Studies in various subtypes of HNSCC have revealed widespread alterations in *HOX* gene expression (**Table 1**). In oral squamous cell carcinoma (OSCC), 18 of the 39 *HOX* genes (*HOXA1*, *HOXA2*, *HOXA3*, *HOXA5*, *HOXA9*, *HOXB3*, *HOXB6*, *HOXB7*, *HOXB9*, *HOXC4*, *HOXC6*, *HOXC8*, *HOXC9*, *HOXC11*, *HOXC13*, *HOXD9*, *HOXD10*, and *HOXD11*) are significantly overexpressed compared with normal mucosal tissue (28). In contrast, a subset of *HOX* genes including *HOXA2* and *HOXA9*, are downregulated in nasopharyngeal carcinoma (NPC) and OSCC, respectively (26, 34, 35). Moreover, certain *HOX* genes, such as *HOXA3*, display stage-dependent expression patterns, being upregulated in dysplastic lesions but downregulated in advanced tumor stages (28, 29).

**Table 1:** Overview of *HOX* gene dysregulation in head and neck squamous cell carcinoma (HNSCC), including reported alterations and associated biological effects.

HOX gene	Type of cancer	Alteration type	Biological effects	References
*HOXA1*	Oral	Upregulation	Proliferation, advanced stage, poor differentiation, perineural invasion, lymph node metastasis, reduced overall survival	(22–25)
*HOXA2*	Nasopharyngeal/Laryngeal	Hypermethylation-associated downregulation	Invasion, metastasis (MMP-9 upregulation)	(26, 27)
*HOXA3*	Oral	Stage-dependent expression: upregulation in dysplasia; downregulation in advanced stages	Tumor progression, advanced stage, poor prognosis	(28, 29)
*HOXA4*	Laryngeal	Downregulation	Altered expression patterns (function not defined)	(27)
*HOXA5*	Oral	Stage-dependent expression: upregulation in dysplasia; downregulation hypermethylation associated in tumor	Better prognosis (high expression), poor survival (low expression)	(30, 31)
*HOXA7*	Oral/Laryngeal	Upregulation	Advanced stage, poor differentiation, vascular invasion, perineural invasion, lymph node and distant metastasis	(27, 32, 33)
*HOXA9*	Oral/Laryngeal	Hypermethylation-associated downregulation (OSCC) Upregulation (LSCC)	Proliferation, tumor aggressiveness (OSCC); tumor-related pathways (LSCC)	(27, 34, 35)
*HOXA10*	Oral/Laryngeal	Upregulation	Proliferation, tumor growth, migration, invasion, advanced stage, poor prognosis	(27, 36)
*HOXA11*	Oral/Laryngeal	Upregulation	Poor prognosis	(24, 27)
*HOXA13*	Oral/Laryngeal	Upregulation	Tumor progression, poor prognosis	(27, 33)
*HOXB5*	Oral	Upregulation	Proliferation, tumor growth, migration, invasion, metastasis, advanced stage	(37, 38)
*HOXB7*	Oral	Upregulation	Proliferation, migration, invasion, apoptosis inhibition, advanced stage, lymph node metastasis, poor survival	(39–41)
*HOXB8*	Oral	Upregulation	Proliferation, migration, invasion, tumor growth, poor prognosis, immunosuppressive microenvironment	(42)
*HOXB9*	Laryngeal	Upregulation	Proliferation, migration, invasion, EMT (Wnt/β-catenin activation)	(43)
*HOXB13*	Nasopharyngeal/Oral	Upregulation	Proliferation, migration, invasion, stemness, tumor growth, poor prognosis	(44, 45)
*HOXC5*	Oral	Upregulation	Early tumorigenesis and progression, increased expression from dysplasia to carcinoma	(46)^*^
*HOXC6*	Oral/Laryngeal/Pharyngeal	Upregulation	Proliferation, migration, apoptosis inhibition (Bcl-2), tumor progression, poor prognosis	(47, 48)
*HOXC8*	Laryngeal	Upregulation	Proliferation, migration, poor differentiation	(49)
*HOXC9*	Oral/Pharyngeal	Upregulation	Migration, EMT, context-dependent proliferation	(50)
*HOXC10*	Oral	Upregulation	Proliferation, invasion, EMT (Wnt signaling), metastasis, poor survival	(50, 51)
*HOXD10*	Oral	Upregulation	Proliferation, migration, reduced overall and disease-specific survival	(52)
*HOXD11*	Oral	Upregulation	Invasion (minimal effect on proliferation)	(52)
*HOXD13*	Oral	Upregulation	Proliferation, migration, poor prognosis	(53)

HNSCC, head and neck squamous cell carcinoma; OSCC, oral squamous cell carcinoma; LSCC, laryngeal squamous cell carcinoma; MMP-9, matrix metalloproteinase-9; EMT, epithelial-mesenchymal transition.

^*^ Evidence derived from a 4-nitroquinoline 1-oxide (4NQO)-induced murine model of oral carcinogenesis and may not fully reflect human HNSCC.

No experimental evidence was identified for the following *HOX* genes in HNSCC during the literature search: *HOXA6*, *HOXB1*, *HOXB2*, *HOXB3*, *HOXB4*, *HOXB6*, *HOXC11*, *HOXC12*, *HOXC13*, *HOXD1*, *HOXD3*, *HOXD4*, *HOXD8*, *HOXD9*, and *HOXD12*.

Integrative bioinformatic analyses of HNSCC datasets, including The Cancer Genome Atlas (TCGA), have identified multiple differentially expressed *HOX* genes associated with tumor stage, HPV infection status, and epigenetic alterations such as DNA methylation, some of which correlate with patient survival (54). These findings suggest that *HOX* gene dysregulation occurs in a coordinated manner, rather than acting as isolated gene-specific events (55).Notably, *HOX* gene expression patterns in HNSCC exhibit marked heterogeneity depending on anatomical subsite and molecular subtype, reflecting their context-dependent roles in tumor biology (56, 57). For example, certain *HOX* genes are preferentially overexpressed in tumors of the oral cavity, larynx, and pharynx, whereas others display reduced expression or potential tumor-suppressive functions in advanced disease stages (24, 58).

Collectively, these findings support the concept that *HOX* genes operate within complex transcriptional networks, in which their oncogenic or tumor-suppressive functions are determined by cellular and molecular context. This coordinated dysregulation represents a prominent feature of HNSCC and provides a foundation for future mechanistic studies aimed at understanding how altered *HOX* activity reshapes transcriptional programs, promotes phenotypic plasticity, and contributes to disease progression.

## 3 Molecular mechanisms and signaling pathways regulated by *HOX* genes in HNSCC

Beyond their aberrant expression, *HOX* genes actively regulate multiple cellular processes central to carcinoma progression, including proliferation, differentiation, apoptosis, angiogenesis, and epithelial-mesenchymal transition (EMT) (59, 60). Rather than acting through a single linear pathway, HOX proteins function as context-dependent transcriptional regulators that modulate complex signaling networks, linking developmental programs with cancer-associated pathways (61).

Aberrant activation of the Wnt/β-catenin signaling pathway is a hallmark of multiple cancers, including HNSCC, where it contributes to tumor growth, invasion, and cellular plasticity (62). Accumulating evidence indicates that several HOX family members can directly or indirectly modulate this pathway and others, thereby, regulating key biological processes such proliferation, apoptosis, differentiation, motility, and angiogenesis (63). For instance, *HOXB5* is frequently overexpressed in HNSCC and has been shown to activate canonical Wnt/β-catenin signaling by binding to the promoter of the epidermal growth factor receptor (EGFR). This interaction enhances tumor cell proliferation, migration, and invasion, and promotes EMT both *in vitro* and *in vivo* (37). Similarly, *HOXB8* upregulation has been implicated in EMT-associated transcriptional programs and metastatic behavior, while also modulating the PI3K/AKT/mTOR signaling axis, to promote cell proliferation and survival. In addition, *HOXB8* suppresses interferon-α (IFN-α) mediated signaling, a pathway critical for antitumor immune responses (42). *HOXB9* has likewise been reported to modulate the Wnt/β-catenin signaling, contributing to increased proliferative capacity and EMT in pharyngeal cancer (43).

In addition to their pro-invasive roles, *HOX* genes can promote tumor cell survival by modulating apoptotic pathways. For example, *HOXC6* induces overexpression of the anti-apoptotic protein Bcl-2 through direct promoter binding, conferring resistance to paclitaxel-induced apoptosis in HNSCC cells (47).

Collectively, these findings highlight the multifaceted roles of *HOX* genes in coordinating key oncogenic processes in HNSCC. However, the level of functional and mechanistic evidence varies considerably among individual *HOX* family members, underscoring the need for further studies to clarify their context specific roles and therapeutic potential.

## 4 Epigenetic, transcriptional, and post-transcriptional regulation of *HOX* genes in HNSCC

*HOX* gene expression is tightly controlled by multiple regulatory layers, including epigenetic, transcriptional, and post-transcriptional mechanisms, which ensure precise spatiotemporal patterns during development (64). In cancer, disruption of these regulatory processes contributes to aberrant *HOX* expression, promoting malignant phenotypes such as uncontrolled proliferation, invasion, and resistance to differentiation. In HNSCC, accumulating evidence indicates that epigenetic remodeling plays a central role in *HOX* gene deregulation (65, 66). These mechanisms, including DNA methylation, histone modifications, chromatin remodeling, and non-coding RNAs, are frequently altered in malignancies and are critical for controlling gene expression (67–69). Given their central role as transcriptional regulators, *HOX* genes are particularly susceptible to these epigenetic alterations, which impact cellular processes such as adhesion, migration, invasion, and survival (70, 71).

Among epigenetic mechanisms, aberrant DNA methylation has been closely linked to *HOX* gene dysregulation across multiple cancer types (72, 73). Genome-wide methylation analyses in OSCC have identified increased CpG methylation within *HOX* gene clusters, correlating with altered transcriptional profiles (74). Subsequent studies have reported methylation levels exceeding 50% across multiple *HOX* genes (75). In OSCC, *HOXA9* is frequently hypermethylated, which is associated with reduced gene expression and increased tumor aggressiveness, including lymph node metastasis (34, 35). These findings are particularly notable, as *HOXA9* has been described as a tumor suppressor in other malignancies, where it limits tumor growth and metastasis, and support the concept that *HOX* genes may not exert tumor-suppressive functions when silenced, frequently through promoter methylation (76). Additional *HOX* genes also exhibit aberrant methylation across squamous cell carcinomas, including *HOXA2* in nasopharyngeal carcinoma (NPC), and *HOXB4* and *HOXC4* in OSCC (26, 77). Collectively, these observations support a role for DNA methylation as a recurrent mechanism contributing to *HOX* deregulation in HNSCC.

In addition to DNA methylation, long non-coding RNAs (lncRNAs) have emerged as important regulators of *HOX* gene expression (78–80). HOTAIR, a lncRNA transcribed from the *HOXC* locus, functions as a molecular scaffold linking RNA-mediated regulation with chromatin remodeling through its interaction with epigenetic complexes such as polycomb repressive complex 2 (PRC2) (81). Increased HOTAIR expression has been associated with tumor size, advanced clinical stage, and poor prognosis, highlighting its potential as a biomarker in HNSCC (82).

At the post-transcriptional level, *HOX* gene expression is further modulated by microRNAs that regulate mRNA stability and translation. MicroRNAs such as miR-196a and miR-10b, which are embedded within *HOX* clusters, exhibit altered expression patterns in tumor tissues and contribute to dysregulated cell proliferation through context-dependent mechanisms (83). In HNSCC, co-expression of *HOXB9* and miR-196a has been associated with increased cell migration and invasion (84). Moreover, miR-196a directly targets *HOXB8* and p27, suppressing their expression in oral cancer (85). Interestingly, *HOXB8* is overexpressed in HNSCC tissues, underscoring the context-dependent effects of miR-196a and the complexity of *HOX* regulatory networks (42).

Finally, *HOX* genes can also act as downstream targets of oncogenic signaling networks driven by cancer-associated mutations. In HNSCC, frequently mutated genes include *TP53*, *FAT1*, and *CDKN2A*, while major dysregulated pathways involve *TP53*, *NOTCH*, *WNT*, and *PI3K* signaling. Integrative oncogenomic analysis have identified transcription factors such as TP53, EP300, MYC, CTCF, and TP63, as key upstream regulators of *HOX* gene expression (23). Notably, tumors harboring TP53 mutations exhibited coordinated upregulation of multiple *HOX* genes, supporting a transcriptionally mediated regulatory relationship. In addition, distinct patterns of *HOX* dysregulation have been observed in *CDKN2A*-mutant and in combined *TP53/FAT1/CDKN2A*-mutant profiles, further highlighting the impact of cancer driver mutations on *HOX*-dependent transcriptional programs.

Overall, *HOX* gene deregulation in HNSCC reflects disruption of a multilayered regulatory networks involving epigenetic remodeling, RNA-mediated control, and oncogenic transcriptional inputs. The convergence of these mechanisms drives aberrant *HOX*-dependent transcriptional programs that promote tumor progression and cellular plasticity. Understanding these integrated regulatory networks provides a framework for identifying *HOX*-associated pathways as potential biomarkers and therapeutic targets in HNSCC.

## 5 HOX protein complexes and regulatory interaction networks in HNSCC

Although HOX proteins function as transcription factors, their intrinsic DNA-binding affinity and specificity are relatively low due to the high structural conservation of the homeodomain and its preference for short, degenerate DNA motifs widely distributed throughout the genome (86–88). Consequently, HOX proteins rarely act alone and instead function as components of multiprotein transcriptional complexes. Interactions with cofactors and additional transcriptional regulators enhance DNA-binding affinity and refine target gene specificity compared with HOX proteins acting independently (89).

A central mechanism of HOX-mediated transcriptional regulation involves the formation of heterodimeric or multimeric complexes with PBX and MEIS cofactors, members of the Three Amino acid Loop Extension (TALE) family of homeodomain proteins (90). These interactions stabilize DNA binding and expand the regulatory capacity of HOX proteins, enabling context-dependent transcriptional activation or repression of target genes (91). This mechanism may partially explain the dual oncogenic or tumor-suppressive roles of HOX family members across different cancers.

Beyond canonical HOX-PBX/MEIS complexes, HOX proteins can engage in diverse protein-protein interactions, including associations with components of the basal transcription machinery, thereby enabling fine-tuning of transcriptional output (92, 93). Although such multimeric HOX-containing complexes have been primarily characterized in developmental systems, particularly in *Drosophila*, these studies demonstrate the capacity of HOX complexes to quantitatively modulate gene expression, favoring either transcriptional activation or repression depending on complex composition (94). Together, these observations support a model in which HOX functional activity arises from dynamic interactions with cofactors and transcriptional components, resulting in regulatory complexes that control gene expression in a spatially and temporally coordinated manner. In cancer cells, aberrant assembly or altered stoichiometry of these complexes may reprogram HOX-dependent transcriptional networks.

Consistent with this framework, emerging evidence indicates that disruption of HOX-PBX interactions hold therapeutic potential across multiple malignancies, including prostate, breast, renal, ovarian, and lung cancers, as well as melanoma, multiple myeloma, and acute myeloid leukemia (87). Although most of this evidence derives from non-head and neck cancers, similar HOX-PBX interaction networks are likely to be relevant in HNSCC (95). In these contexts, structural or functional alterations of HOX-containing complexes may rewire transcriptional programs, promoting oncogenic pathways associated with cell survival, invasion, and metastasis.

## 6 Clinical relevance of *HOX* genes in HNSCC: prognostic and therapeutic implications

Translating molecular insights into clinical applications remains a major challenge in HNSCC. Given their central role in developmental regulation and oncogenic transcriptional networks, *HOX* genes have emerged as promising candidates for clinical stratification, offering opportunities for improved prognostic assessment and therapeutic intervention (72). Increasing evidence indicates that alterations in *HOX* gene expression not only reflect tumor biology but also correlate with disease stage, metastatic potential, and patient outcomes (96, 97).

Accurate characterization of disease stage is essential for prognosis in HNSCC. In this context, *HOX* gene expression profiles have gained attention as potential biomarkers. Lymph node metastasis represents a key prognostic indicator in HNSCC (98). Elevated expression of *HOXA1* and *HOXB7* has been associated with lymph node metastasis, supporting their link to aggressive disease behavior (22, 41). Similarly, *HOXB9* upregulation has been reported across multiple cancer types and associated with poor prognosis and altered immune responses (99). In HNSCC, multi-omics analyses have further shown that *HOXB8* overexpression promotes tumor progression and correlates with adverse clinical outcomes (42).

Beyond prognosis, *HOX* gene expression patterns may also inform disease staging and early detection. For example, *HOXC5* expression is significantly higher in dysplastic lesions compared with hyperplastic oral tissues, suggesting its potential utility in distinguishing early disease stages (46). Additionally, *HOXD10* has been reported to reduce cell invasion while enhancing proliferation, adhesion, and migration, indicating a context-dependent role during early tumor development and supporting its potential value as an early-stage biomarker (100).

Overall, these findings highlight the clinical potential of *HOX* genes as biomarkers for prognosis, disease stratification, and early detection in HNSCC. However, their translation into clinical practice remains limited, underscoring the need for further validation in large, well-characterized patient cohorts. Future studies integrating multi-omics approaches and functional validation will be essential to establish *HOX* genes as reliable clinical tools and to explore their potential as therapeutic targets.

## 7 Conclusions and perspectives

*HOX* genes emerge as central regulators of transcriptional programs that underline both normal development and malignant transformation. In HNSCC, their dysregulation reflects the convergence of epigenetic remodeling, RNA-mediated regulation, genetic alterations, and aberrant assembly of transcriptional complexes, collectively driving tumor initiation, progression, and phenotypic plasticity. Rather than acting as isolated factors, HOX proteins function within dynamic regulatory networks that integrate developmental cues with oncogenic signaling pathways. Accumulating evidence highlights the clinical relevance of *HOX* gene expression and associated regulatory networks as biomarkers for disease stratification, prognosis, and early detection. Although direct targeting of individual *HOX* genes remains challenging due to functional redundancy and context-dependent effects, emerging therapeutic strategies aim to disrupt oncogenic HOX protein-protein interactions, such as HOX-PBX complexes, or to modulate upstream regulatory mechanisms, including epigenetic modifiers and non-coding RNAs. While these approaches remain largely in preclinical stages, they represent promising avenues for selectively targeting *HOX*-driven oncogenic programs in HNSCC. Continued integrative and functional studies will be essential to translate insights about *HOX* biology into clinically useful tools, ultimately enabling advancements in precision oncology strategies for patients with HNSCC.
